# Repurposing MK-8245 as a Quorum Sensing Inhibitor to Suppress Virulence and Potentiate Antibiotic Activity in *Pseudomonas aeruginosa*

**DOI:** 10.3390/antibiotics14111116

**Published:** 2025-11-05

**Authors:** Giulia Bernabè, Giovanni Marzaro, Mahmoud Elsayed Mosaad Shalata, Daniela Iosob, Valentina Inglima, Massimo Bellato, Ignazio Castagliuolo, Paola Brun

**Affiliations:** 1 Department of Molecular Medicine, University of Padua, 35121 Padua, Italy; mahmoudelsayedmosaad.shalata@phd.unipd.it (M.E.M.S.); daniela.iosob@studenti.unipd.it (D.I.); valentina.inglima@studenti.unipd.it (V.I.); ignazio.castagliuolo@unipd.it (I.C.); paola.brun.1@unipd.it (P.B.); 2 Department of Diagnostics and Public Health, University of Verona, 37134 Verona, Italy; giovanni.marzaro@univr.it; 3 Department of Information Engineering, University of Padua, 35131 Padua, Italy; massimo.bellato@unipd.it

**Keywords:** biofilm, antimicrobial resistance, virulence, quorum sensing inhibitors, molecular docking

## Abstract

**Background/Objectives**: The rise in multidrug-resistant pathogens such as *Pseudomonas aeruginosa* (PA), coupled with declining antibiotic development, underscores the need for innovative therapeutic strategies. Repurposing approved drugs provides advantages of safety and rapid development. Since quorum sensing (QS) controls key virulence traits in PA, targeting this pathway represents a promising antivirulence approach. This study aimed to identify and repurpose existing drugs as QS inhibitors. **Methods**: An in silico docking screen of 3000 FDA-approved or clinically tested compounds was performed against the C4-HSL receptor RhlR. Seventeen candidates were tested in the laboratory strain PAO1 for lactone-dependent signaling inhibition. The most active compound, MK-8245, was further evaluated for effects on growth, cytotoxicity, lactone release, biofilm formation, pyocyanin, elastase, rhamnolipids, and swarming motility. Its activity was also assessed in 20 clinical PA isolates. **Results**: MK-8245 (40 µM) reduced QS-regulated gene expression by ~60% without affecting viability. In PAO1, it inhibited rhamnolipids (60%), pyocyanin (40%), elastase (25%), biofilm formation, and swarming motility (25%). MK-8245 also enhanced the efficacy of imipenem against biofilms. In clinical isolates, it consistently decreased lactone release (~60%), pyocyanin (~50%), rhamnolipids (~40%), biofilm formation (~30%), and swarming motility (~25%). **Conclusions**: MK-8245 emerges as a promising antivirulence candidate against *P. aeruginosa*. By disrupting QS signaling and impairing multiple virulence factors, it attenuates pathogenicity without bactericidal pressure. Its synergy with standard antibiotics and consistent activity in clinical isolates highlight its translational potential and warrant further preclinical evaluation.

## 1. Introduction

*Pseudomonas aeruginosa* is a ubiquitous Gram-negative opportunistic pathogen responsible for a broad spectrum of acute and chronic infections, particularly in immunocompromised patients such as those with cystic fibrosis, severe burns, or undergoing invasive medical procedures [1,2]. Its clinical relevance is amplified by intrinsic resistance to multiple antibiotic classes and the rapid acquisition of additional resistance mechanisms through horizontal gene transfer and chromosomal mutations, making it one of the most challenging multidrug-resistant (MDR) pathogens worldwide [1,3]. The combined ability to resist antimicrobials, form biofilms, and secrete a wide array of virulence factors contributes to the persistence and recalcitrance of *P. aeruginosa* infections in healthcare settings.

The urgency to develop alternative therapeutic strategies is underscored by the global rise in antimicrobial resistance (AMR). The World Health Organization (WHO) has designated carbapenem-resistant *P. aeruginosa* as a “critical priority” pathogen requiring novel treatment options [4]. Global surveillance reports carbapenem resistance rates of 20–40%, with the highest prevalence in Asia and Eastern Europe [4]. In the European Union/European Economic Area (EU/EEA), approximately 15% of *P. aeruginosa* isolates are resistant to at least one carbapenem, with markedly higher levels in Romania, Greece, and Italy [5]. In Italy, national surveillance conducted by the Istituto Superiore di Sanità (ISS) documented resistance rates to meropenem and imipenem above 16% in 2022, with MDR strains accounting for over 12% of isolates—particularly in intensive care units, where invasive devices and prolonged hospitalizations favor chronic colonization and infection [6].

A central determinant of *P. aeruginosa* pathogenicity is quorum sensing (QS), a complex cell density-dependent regulatory system that controls the expression of genes involved in virulence, biofilm formation, motility, and immune evasion [7]. The QS network in *P. aeruginosa* comprises four interconnected circuits: Las, Rhl, Pqs, and Iqs. Traditionally, the Las system, governed by LasR and its autoinducer *N*-(3-oxododecanoyl)-L-homoserine lactone (3-oxo-C12-HSL), was considered to occupy the top of the hierarchy, regulating genes such as *lasI* and *lasB* [8]. However, accumulating evidence indicates that RhlR plays a central role, not only controlling rhamnolipid (throughout *rhlA* gene) and pyocyanin (*phzA1* gene) production through its signal *N*-butyryl-L-homoserine lactone (C4-HSL) but also functioning as the primary QS regulator in LasR-deficient clinical isolates [9,10]. Importantly, the Rhl and Pqs systems are tightly interconnected: quinolone-derived signals such as PQS (Pseudomonas Quinolone Signal) modulate RhlR activity and sustain virulent gene expression, thereby enabling adaptive QS responses in diverse infection niches [11].

Targeting QS has gained attention as an antivirulence strategy, as it suppresses pathogenicity without directly affecting bacterial viability. Quorum sensing inhibitors (QSIs) can attenuate virulence and biofilm formation while reducing selective pressure for resistance development, and they may potentiate the activity of conventional antibiotics [12,13]. Among the most promising approaches is drug repurposing, which leverages existing data on safety, pharmacokinetics, and toxicity to shorten development timelines and reduce costs compared with de novo drug discovery [14,15,16]. In this study, candidate compounds were identified through an in silico docking screening of 3000 FDA-approved or clinically tested molecules against the C4-HSL receptor RhlR, a central regulator of quorum sensing in *P. aeruginosa*. The docking workflow was based on the model reported by Bernabè et al. [17], which delineates the ligand-binding site that recognizes the native autoinducer C4-HSL. This model was employed to virtually screen compounds predicted to occupy the same binding pocket and thereby act as competitive antagonists, preventing autoinducer engagement and subsequent quorum-sensing activation. From this screening, seventeen top-scoring compounds were experimentally tested against the PAO1 laboratory strain for inhibition of lactone-dependent signaling. Among the tested compounds, MK-8425 emerged as the most effective inhibitor of RhlR-dependent transcriptional activity and was therefore selected for further characterization. MK-8245 is a compound developed initially for metabolic disorders and previously tested in clinical trials for type 2 diabetes. In this study, we evaluated the repurposing potential of MK-8245 as a novel QSI. Indeed, we demonstrate that MK-8245 interferes with QS-regulated gene expression, attenuates virulence factor production, impairs biofilm formation, and enhances antibiotic efficacy against both laboratory and clinical *P. aeruginosa* isolates. These findings highlight the feasibility of repurposing clinically evaluated drugs as a rapid and effective strategy to combat MDR bacterial infections.

## 2. Results

### 2.1. Screening of Candidate Compounds for Inhibition of the Rhl Quorum Sensing Pathway

**Figure 1:**
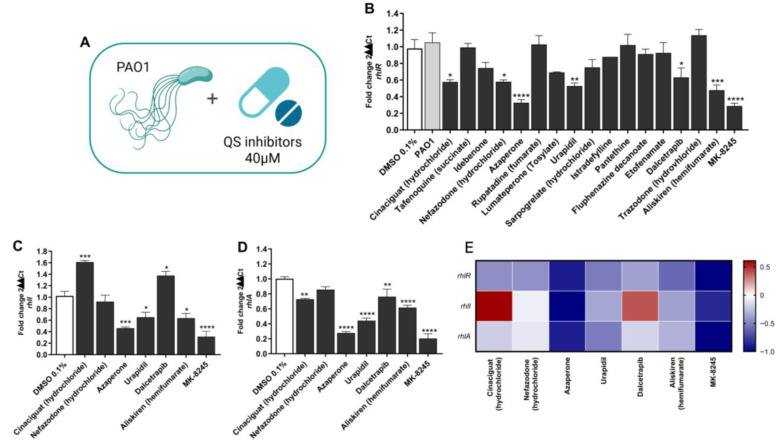
Screening of FDA-approved and clinically evaluated compounds for inhibition of Rhl quorum sensing in *Pseudomonas aeruginosa* PAO1. (**A**) Schematic representation of the screening strategy, in which PAO1 was cultured with candidate quorum sensing inhibitors (QSIs) at 40 µM. (**B**) Initial screening results showing the effect of 15 compounds on *rhlR* expression, assessed by qRT-PCR in PAO1 planktonic cultures. Expression values are presented as fold change relative to untreated controls (set to 1.0). (**C**,**D**) Validation of selected hits, showing relative expression of *rhlI* (**C**) and *rhlA* (**D**). (**E**) Heatmap summarizing the inhibitory effects of the most active compounds across *rhlR*, *rhlI*, and *rhlA* expression. MK-8245 displayed the most potent inhibitory activity among all tested molecules. Data are expressed as mean ± SD of three independent experiments performed in triplicate. Statistical significance was determined by two-way ANOVA with Bonferroni’s post hoc test (* *p* < 0.05; ** *p* < 0.01; *** *p* < 0.001; **** *p* < 0.0001 vs. DMSO 0.1%).

To identify compounds capable of interfering with QS in *P. aeruginosa*, we assessed the expression of genes regulated by the Rhl pathway using qRT-PCR in PAO1 planktonic cultures exposed to a panel of FDA-approved or clinically evaluated drugs (40 µM; Figure 1A).

As shown in Figure 1B, several compounds reduced *rhlR* mRNA level compared with the DMSO control. Among these, MK-8245 exhibited the strongest inhibitory effect, with a ~80% reduction in *rhlR* expression (*p* < 0.0001). Other compounds with significant activity included nefazodone, cinaciguat, azaperone, and aliskiren, although their effects were less pronounced.

Consistent with these findings, MK-8245 also markedly decreased the expression of *rhlI* and *rhlA*, which encode the autoinducer synthase and rhamnosyltransferase subunit A, respectively (Figure 1C,D).

The overall inhibitory profile of the most active compounds across the three target genes is summarized in the heatmap (Figure 1E). MK-8245 consistently showed the most potent activity, leading us to select it for subsequent functional characterization.

### 2.2. MK-8245 Interacts with RhlR and Reduces QS Signal Molecule Production Without Affecting Growth

**Figure 2:**
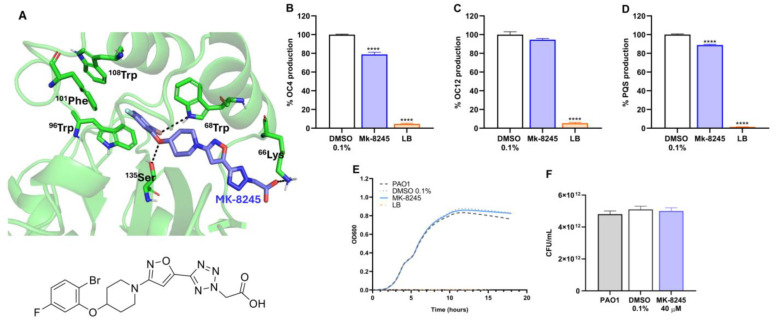
Molecular docking of MK-8245 to RhlR and its effect on autoinducer production and bacterial growth in *P. aeruginosa* PAO1. (**A**) Docking model showing the predicted interaction of MK-8245 with the RhlR binding pocket. Key amino acid residues involved in hydrogen bonding and hydrophobic interactions are indicated. The chemical structure of MK-8245 is also shown. (**B**–**D**) Quantification of AHL signal production in PAO1 treated with MK-8245 (40 µM) or vehicle (DMSO 0.1%). Results were obtained using biosensor strains kindly provided by Prof. Rampioni (17): (**B**) C4-HSL, (**C**) 3-oxo-C12-HSL, and (**D**) PQS production. (**E**) Growth curves of PAO1 in the presence or absence of MK-8245 (40 µM) compared to untreated and LB controls, showing no impact on bacterial growth kinetics. (**F**) Colony-forming unit (CFU) counts after 24 h of culture confirm the absence of bactericidal or bacteriostatic effects. Data are presented as mean ± SD from three independent experiments performed in triplicate. Statistical analysis was conducted using two-way ANOVA with Bonferroni’s post hoc test (**** *p* < 0.0001).

Molecular docking simulations predicted that MK-8245 interacts with the RhlR binding pocket through hydrogen bonds with Lys66 and Ser135, and hydrophobic interactions with Trp68, Trp96, Trp101, and Trp108 (Figure 2A). As the mentioned residues form the binding pocket for C4HSL, the binding mode suggests potential interference with acyl-homoserine lactone (AHL) recognition.

Consistent with the in silico prediction, functional assays using *P. aeruginosa* biosensor strains demonstrated a significant reduction in C4-HSL and PQS production upon treatment with MK-8245 (40 µM) compared to the DMSO control (*p* < 0.0001, Figure 2B). In contrast, the compound did not markedly affect 3-oxo-C12-HSL levels, indicating a selective interference with the Rhl system (Figure 2C,D).

To exclude the possibility that these effects were due to impaired bacterial growth, we evaluated *P. aeruginosa* PAO1 proliferation in the presence of MK-8245. Growth kinetics monitored over 20 h (Figure 2E) and colony counts after 24 h (Figure 2F) showed no significant differences between treated and untreated cultures, confirming that MK-8245 does not exert bacteriostatic or bactericidal effects at the tested concentration. The calibration curves used for quantification of C4-HSL, 3-oxo-C12-HSL, and PQS signals are provided in Figure S1.

### 2.3. MK-8245 Reduces Virulence-Associated Factor in P. aeruginosa PAO1

To determine the potency of MK-8245, dose–response analyses were performed for key virulence-related phenotypes. MK-8245 inhibited pyocyanin, luminescence, and rhamnolipid production with IC_50_ values of 58.4, 35.0, and 29.2 µM, respectively (Table S1 and Figure S2). The IC_50_ for swarming motility could not be reliably. The mean IC_50_ value of approximately 40 µM was therefore selected for subsequent experiments as the lowest concentration producing maximal inhibitory effects.

**Figure 3:**
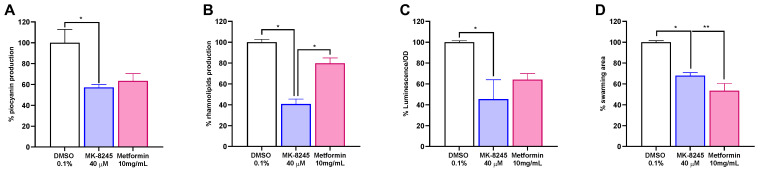
MK-8245 reduces the production of quorum sensing–regulated virulence factors in *Pseudomonas aeruginosa* PAO1. (**A**) Pyocyanin levels measured after 24 h of culture, showing significant reduction in the presence of MK-8245 (40 µM). (**B**) Rhamnolipid quantification following 24 h incubation demonstrates marked inhibition by MK-8245. (**C**) Bioluminescence assay with *Vibrio harveyi* BB120 reveals decreased secretion of acyl-homoserine lactones (AHLs) in MK-8245–treated cultures. (**D**) Swarming motility assays performed on semi-solid agar plates indicate reduced motility area in the presence of MK-8245. All results are normalized to untreated PAO1 (DMSO 0.1%), which was set as 100%. Metformin (10 mg/mL) was included as a positive control, as previously reported to inhibit quorum-sensing–regulated virulence factors. Data are reported as the mean ± SD from three independent experiments performed in triplicate. Statistical analysis was conducted using one-way ANOVA followed by multiple comparisons test (* *p* < 0.05; ** *p* < 0.01).

The effect of MK-8245 on major virulence factors of *P. aeruginosa* PAO1 was then evaluated at 40 µM and compared with the reference antidiabetic drug metformin, used as a positive control. As shown in Figure 3, both compounds reduced virulence-associated traits, although MK-8245 consistently displayed stronger inhibition. Specifically, MK-8245 decreased pyocyanin production by approximately 40% compared with the DMSO control (Figure 3A), and markedly reduced rhamnolipid secretion by about 60% (Figure 3B). Lactone production, quantified using *Vibrio harveyi*–based biosensors, was also strongly impaired, with a ~60% reduction relative to controls (Figure 3C). Finally, MK-8245 decreased swarming motility, resulting in a ~25% reduction in migration area compared with untreated PAO1 (Figure 3D).

### 2.4. MK-8245 Inhibits Biofilm Formation and Enhances Antibiotic Activity Against Pre-Formed Biofilms

**Figure 4:**
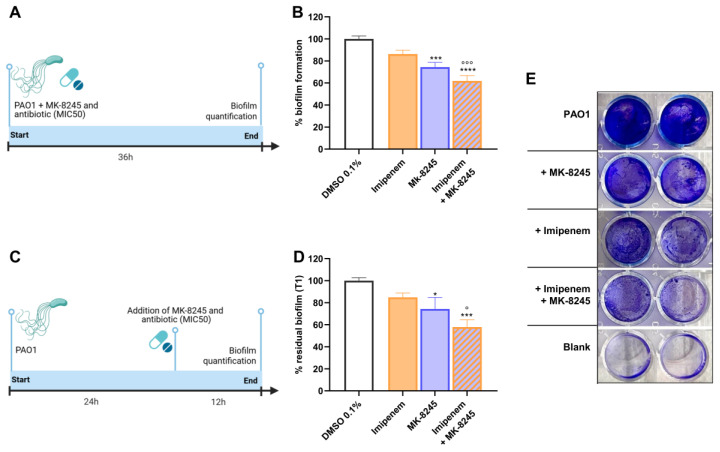
MK-8245 enhances the antibiofilm activity of imipenem against *Pseudomonas aeruginosa* PAO1. (**A**) Experimental design for biofilm inhibition assays: MK-8245 (40 µM), imipenem (MIC_50_), or the combination were added at the time of inoculation (T0), and biofilm biomass was quantified after 36 h. (**B**) Quantification of biofilm biomass by crystal violet staining shows a significant reduction with MK-8245 or imipenem alone, and an additive effect with the combination. (**C**) Experimental design for biofilm eradication assays: MK-8245 (40 µM), imipenem (MIC_50_), or the combination were added after 24 h to pre-formed biofilms (T1), and biomass was quantified at 36 h. (**D**) Residual biomass of established biofilms after treatment, showing that MK-8245 enhanced the antibiofilm effect of imipenem. (**E**) Representative images of stained biofilms corresponding to the conditions tested. Data are expressed as mean ± SD from three independent experiments performed in triplicate. Statistical analysis was performed using two-way ANOVA with Bonferroni’s post hoc test (* *p* < 0.05; *** *p* < 0.001; **** *p* < 0.0001 vs. DMSO 0.1%; ° *p* < 0.05; °°° *p* < 0.001 vs. Imipenem).

The effect of MK-8245 on biofilm formation and maintenance was evaluated in PAO1 using the crystal violet staining method. When added at the time of bacterial inoculation (T0), MK-8245 significantly reduced biofilm biomass after 36 h of incubation (Figure 4A,B). The combination of MK-8245 with imipenem (MIC_50_) further enhanced this effect, resulting in the most potent inhibition of biofilm formation.

When treatments were applied to pre-formed biofilms after 24 h of incubation (T1), both MK-8245 and imipenem alone produced a measurable reduction in residual biofilm biomass; however, the combination achieved the most significant decrease (Figure 4C,D). Representative images of crystal violet-stained wells illustrate the visible reduction in biofilm biomass under combined treatment compared with single treatments or controls (Figure 4E).

### 2.5. MK-8245 Improves Survival in the Galleria mellonella Infection Model

**Figure 5:**
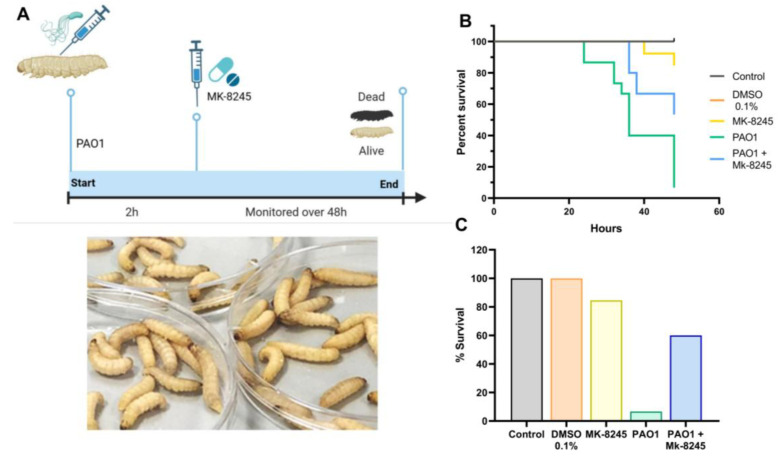
MK-8245 improves survival in a *Galleria mellonella* infection model of *Pseudomonas aeruginosa* PAO1. (**A**) Experimental design of the in vivo assay. Larvae were infected with PAO1 (10^6^ CFU/larva) and, after 2 h, treated with MK-8245 (40 µM) or vehicle (DMSO 0.1%). Survival was monitored over 48 h. Representative images of larvae used in the experiments are shown. (**B**) Kaplan–Meier survival curves demonstrating that MK-8245 significantly improved larval survival compared to untreated PAO1-infected controls. (**C**) Percentage survival at 48 h for each group. MK-8245 alone and vehicle controls showed no toxicity, while treatment of infected larvae with MK-8245 partially restored survival compared to PAO1-infected controls. Data are pooled from three independent experiments with 30–50 larvae per group. Statistical analysis was performed using the log-rank (Mantel–Cox) test (*p* < 0.05).

The protective effect of MK-8245 against *P. aeruginosa* PAO1 infection was evaluated in the *Galleria mellonella* larva model (Figure 5A). Larvae infected with PAO1 exhibited high mortality, with survival dropping to <20% within 48 h (Figure 5B). In contrast, larvae treated with MK-8245 after infection demonstrated significantly improved survival compared with untreated infected controls. Larvae injected with vehicle alone (DMSO 0.1%) or with MK-8245 alone showed no toxicity within the experimental timeframe (Figure 5B). Kaplan–Meier survival curves showed a delay in mortality in the group infected with POA1 and treated with MK-8245, with ~60% of larvae surviving at 48 h (Figure 5B). Endpoint analysis confirmed this protective effect, with markedly higher survival in infected MK-8245-treated larvae compared with those infected with PAO1 but not treated (Figure 5C). No toxicity was observed in larvae treated with MK-8245 or DMSO vehicle controls in the absence of infection.

### 2.6. MK-8245 Reduces Virulence Factor Production in Clinical P. aeruginosa Isolates

**Figure 6:**
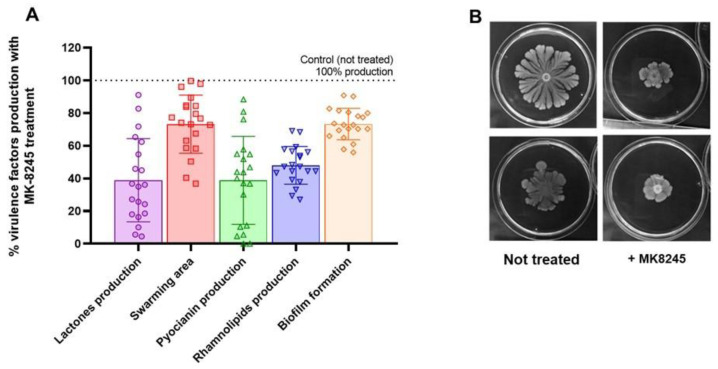
MK-8245 attenuates virulence factor production in clinical isolates of *Pseudomonas aeruginosa*. (**A**) Box plots summarize the effect of MK-8245 (40 µM) on lactone release, swarming motility, and pyocyanin production in 20 clinical isolates compared to untreated controls (set at 100%). MK-8245 treatment significantly reduced all three parameters, with median reductions of approximately 60% for lactones, 25% for swarming motility, 60% for pyocyanin, 50% for rhamnolipids, and 25% for biofilm. (**B**) Representative swarming assays showing the reduction in migration area following MK-8245 treatment compared to untreated controls. Data are presented as mean ± SD from three independent experiments.

To evaluate the activity beyond the laboratory PAO1 strain, MK-8245 was tested on 20 *P. aeruginosa* clinical isolates obtained from respiratory tract infections. Treatment with MK-8245 (40 µM) significantly reduced the production of multiple QS-regulated virulence factors (Figure 6).

On average, lactone release was reduced by ~60%, pyocyanin production by ~50%, rhamnolipid production by ~40%, biofilm formation by ~30%, and swarming motility by ~25%. These inhibitory effects were consistently observed across the clinical isolate panel. Representative swarming assays illustrate the marked reduction in motility upon MK-8245 treatment (Figure 6B).

## 3. Discussion

The increasing prevalence of multidrug-resistant (MDR) *Pseudomonas aeruginosa* highlights the urgent need for alternative therapeutic strategies beyond conventional antibiotics. In recent years, drug repurposing has emerged as a pragmatic approach to accelerate the discovery of antivirulence agents by leveraging the established pharmacological and safety profiles of FDA-approved compounds [15,18,19].

Several screening studies have identified non-antibiotic drugs with quorum-sensing (QS) inhibitory activity, including anti-inflammatory and antipsychotic agents [14]. Recent studies have explored the use of antidiabetic drugs as quorum-sensing (QS) inhibitors in *Pseudomonas aeruginosa*. The dipeptidyl peptidase-4 (DPP-4) inhibitors sitagliptin, vildagliptin, and linagliptin, as well as the biguanide metformin, were shown to reduce QS-regulated virulence traits—such as pyocyanin, protease production, motility, and biofilm formation—mainly by downregulating the *las*, *rhl*, and *pqs* systems [20,21,22]. Mechanistically, these agents act as QS antagonists that compete with native acyl-homoserine lactones for binding to the LasR and RhlR receptors, thereby suppressing QS-dependent gene expression and virulence without affecting bacterial growth [20,21,22]. In contrast to previous studies, our work identified MK-8245, an antidiabetic compound developed initially for metabolic disorders, as a potent and selective RhlR antagonist, with mechanistic and experimental evidence supporting its quorum-sensing inhibitory activity in both laboratory and clinical isolates of *Pseudomonas aeruginosa*.

Using a previously developed pharmacophore-guided docking model (17), we provide mechanistic evidence for RhlR binding and show that MK-8245 not only inhibits multiple QS-regulated virulence factors and biofilm formation but also enhances antibiotic activity, retains efficacy across diverse clinical isolates, and confers significant protection in an in vivo *Galleria mellonella* model. The *G. mellonella* infection model surely has some limitations, including the inter-batch variability regarding the larval immune maturity and the absence of mammalian adaptive immune responses. However, we believe that our findings highlight MK-8245 as a promising repurposed drug candidate with broader, more translational antivirulence potential than previously tested antidiabetic agents.

Considering our previously developed pharmacophore model of the RhlR receptor [17], we demonstrated that MK-8245 interacts with key residues within the RhlR binding site, including Lys66, Ser135, and multiple conserved tryptophans, suggesting interference with ligand recognition and transcriptional activation. These interactions occur within the ligand-binding pocket normally occupied by the native autoinducer *N*-butanoyl-L-homoserine lactone (C4-HSL) [17]. By occupying this site, MK-8245 is predicted to hinder the conformational rearrangements required for RhlR dimerization and DNA binding, thereby preventing activation of QS-regulated genes such as *rhlI* and *rhlA*, which are responsible for rhamnolipid biosynthesis and biofilm matrix development [10,23]. This mechanistic link between receptor antagonism and phenotypic output explains the observed reduction in biofilm formation and virulence factor production in MK-8245-treated cultures.

Importantly, the biosensor assays confirmed the functional consequence of this interaction: MK-8245 markedly reduced production of C4-HSL and PQS while leaving 3-oxo-C12-HSL levels largely unaffected. This differential profile strongly supports the notion that MK-8245 selectively targets the Rhl system rather than nonspecifically suppressing quorum sensing. Such pathway selectivity is consistent with the hierarchical architecture of QS in *P. aeruginosa*, where Rhl and Pqs circuits are tightly interconnected and downstream of Las regulation [8,24]. Prior in silico studies have identified small molecules predicted to bind QS regulators, yet many lacked functional validation or displayed broad inhibitory activity across multiple circuits [8,25]. In contrast, the combined computational and experimental evidence presented here demonstrates not only that MK-8245 fits into the RhlR pharmacophore but also that its effects align with the predicted target specificity, thereby validating the pharmacophore-guided docking approach as a robust strategy for identifying pathway-specific QS inhibitors.

Beyond individual virulence traits, one of the most clinically relevant findings of our study is the ability of MK-8245 to interfere with biofilm formation and, significantly, potentiate the activity of conventional antibiotics against biofilm-embedded cells. Biofilm-associated tolerance remains the most critical obstacle to treating *P. aeruginosa* infections, as biofilms confer protection against host immunity and drastically reduce antibiotic efficacy [26,27]. Previous studies have reported that QS inhibitors can enhance the penetration or activity of antibiotics within biofilms. For example, Brackman and Coenye demonstrated that azithromycin and other QSIs synergized with tobramycin or ciprofloxacin in reducing biofilm biomass [28], while Baldelli et al. showed that the repurposed drug clofoctol increased the susceptibility of *P. aeruginosa* biofilms to multiple antibiotics [29]. However, many of these compounds remain experimental or lack safety profiles suitable for clinical development. Our data show that MK-8245 not only reduces biofilm biomass when administered at the time of inoculation but also disrupts pre-formed biofilms. This condition is much more stringent and clinically relevant. Moreover, the combination of MK-8245 with imipenem yielded the most substantial reduction in biofilm biomass, surpassing the effects of either agent alone. This dual ability to inhibit biofilm development and to sensitize established biofilms to antibiotics places MK-8245 among a select group of molecules with demonstrated antibiofilm–antibiotic synergic effects. Importantly, unlike many experimental QSIs, MK-8245 has undergone prior clinical evaluation as an antidiabetic drug, providing a favorable starting point for translational development. Together, these findings suggest that targeting RhlR with MK-8245 may represent a viable strategy to weaken the biofilm barrier and restore antibiotic efficacy in difficult-to-treat *P. aeruginosa* infections.

Clinical *P. aeruginosa* strains are genetically and phenotypically heterogeneous, with frequent rewiring or attenuation of QS pathways, particularly LasR loss-of-function variants in cystic fibrosis (CF) airway isolates and wound isolates, while Rhl and Pqs circuits often remain operative or become partially uncoupled from Las [9,30,31,32]. This variability has historically limited the generalizability of antivirulence agents discovered in PAO1, as several candidates showed diminished or inconsistent activity across patient-derived strains (strain-to-strain variability in clinical isolates limits the generalizability of PAO1-only antivirulence studies) [33,34]. In contrast, MK-8245 retained activity in a panel of 20 respiratory clinical isolates, reducing lactone release, pyocyanin production, and swarming motility, indicating that targeting RhlR can be effective despite QS diversity in clinical settings. Prior studies have evaluated QS interference in non-laboratory strains, but many focused on single phenotypes or a small number of isolates and often reported strain-dependent outcomes for biofilm or pyocyanin inhibition (e.g., variable responses in CF and wound isolates) [14,35,36]. By demonstrating consistent inhibition of multiple QS-regulated outputs across a broader isolate set—and complementing these data with in vivo protection in the *Galleria mellonella* model—our results argue that Rhl-centric inhibition is a robust strategy with translational promise beyond PAO1. Notably, showing antivirulence efficacy in clinical isolates aligns with accumulating evidence that successful QS-targeted approaches must accommodate the QS plasticity and niche-specific evolution characteristic of *P. aeruginosa* in real infections [32,37,38].

Taken together, these findings position MK-8245 as a promising candidate for antivirulence therapy against *P. aeruginosa*. By specifically targeting QS pathways, MK-8245 attenuates pathogenic traits, possibly without imposing direct selective pressure for resistance. Limitations of the present work include the absence of mammalian infection models and pharmacokinetic assessments, which will be required to evaluate clinical feasibility. Nonetheless, this study contributes to the growing body of evidence that repurposing FDA-approved drugs or clinically tested compounds for antivirulence applications represents a feasible and impactful strategy to expand the antimicrobial pipeline.

## 4. Materials and Methods

### 4.1. Bacterial Strains and Growth Conditions

*P. aeruginosa* (Schroeter 1872) Migula 1900, strain DSM 50071ᵀ (also cataloged as CCUG241 and PAO1 [39] was cultivated in Luria–Bertani (LB) broth.

To assess acyl-homoserine lactone (AHL) production, bioluminescence assays were performed using two *Vibrio harveyi* strains, as described by Henke and Bassler (2004) [40]: *V. harveyi* BB120 (positive for HAI-1 and AI-2, but lacking CAI-1 response) and JAF548, the latter serving as a negative control due to constitutive LuxO activation, which prevents QS-dependent luminescence [41]. Both strains were maintained at 30 °C in Marine Agar or Broth (Difco) at pH 7.0. Experiments were carried out using Autoinducer Bioassay (AB) medium prepared according to Vilchez et al. (2007) [42].

### 4.2. Clinical Isolates

A total of 20 *P. aeruginosa* strains were isolated from sputum samples of patients with lower respiratory tract infections and processed at the Clinical Microbiology Laboratory of Padua University Hospital. Species identification was performed by MALDI-TOF mass spectrometry, and antimicrobial susceptibility testing was conducted by broth microdilution (BMD) using the Sensititre™ Gram Negative Susceptibility Testing Plate (Trek Diagnostic System, Thermo Fisher Scientific, Waltham, MA, USA), in accordance with the 2024 EUCAST breakpoints.

### 4.3. Compound Selection

Building on our previously developed in silico molecular docking model targeting the RhlR binding site of the C4-HSL receptor, we screened a library of 3000 compounds, including FDA-approved drugs and molecules under clinical evaluation, using the same approach as in our previous paper [17]. Briefly, the structure of RhlR was retrieved from the Protein Data Bank (PDB-ID: 8b4a), whereas compound structures were prepared starting from the SMILES code reported in the “FDA-approved” and “investigational compounds” library available from MedChemExpress. Compounds were docked using the software AutoDock Vina version 1.2.0 [43]. The docking box (25 × 25 × 25 angstrom dimension) was centered on the C4-HSL binding pocket. For each compound, 10 poses were generated with the “exhaustiveness” parameter set to 30. Compound selection was based on both docking score and adherence with identified pharmacophoric features, i.e., one hydrogen bond with ^68^Trp, one additional hydrogen bond with a residue of the AHL binding pocket, one arene-arene interaction with a residue in the AHL binding pocket [17]. Initial screening was performed by qRT-PCR, assessing the ability of candidate compounds to reduce the expression of genes involved in the Rhl quorum sensing pathway in PAO1 planktonic cultures. Compounds showing significant downregulation of Rhl-regulated genes were selected for subsequent functional assays. During this process, antitumor agents were excluded due to their high toxicity, and classical antimicrobial drugs were discarded, as the study specifically aimed to identify antivirulence candidates rather than bactericidal agents. The complete list of compounds tested, including their names, sources, purity, and CAS numbers, is provided in Table 1. Compound MK-8245 was purchased from MedChemExpress (CAS Number: 1030612-90-80; Catalog Number: HY-13070; Purity declared: 99.04%; SMILES CODE: O=C(CN1N=C(N=N1)C2=CC(N3CCC(CC3)OC4=CC(F)=CC=C4Br)=NO2)O).

### 4.4. RNA Isolation and Gene Expression Analysis by qRT-PCR

PAO1 cultures were grown in LB broth at 37 °C for 24 h in the presence or absence of the selected compound. Cells were pelleted by centrifugation, and total RNA was extracted using the GRS Total RNA Kit–Bacteria (#GK16.0100, GRISP Research Solution, Porto, Portugal), combining mechanical and enzymatic lysis according to the manufacturer’s instructions. Residual genomic DNA was removed by DNase I treatment. RNA quality was confirmed spectrophotometrically (A260/A280 ratio 1.8–2.0).

Quantitative real-time PCR (RT-qPCR) was then performed directly on RNA samples using the iScript One-Step RT-PCR Kit with SYBR Green (Bio-Rad, Hercules, CA, USA) on a Bio-Rad thermal cycler. Gene-specific primers are listed in Table 2, with *proC* serving as the housekeeping control. Relative gene expression was calculated using the 2^−ΔΔCt^ method, with untreated PAO1 cultures as reference. All reactions were run in triplicate.

Preliminary time-course experiments indicated that expression of most AHL-regulated genes peaked after 24 h of culture [17], and this time point was therefore selected for all subsequent analyses.

### 4.5. Growth Inhibition Assay

MK-8245, the compound identified as the most potent RhlR inhibitor in PAO1, was subsequently evaluated for potential cytotoxic effects. Overnight PAO1 cultures were centrifuged, resuspended in LB broth to ~10^8^ CFU/mL, and diluted to a final inoculum of 1 × 10^6^ CFU in 100 µL per well. Cultures were distributed into sterile 96-well microtiter plates and incubated at 37 °C with MK-8245, DMSO (0.1%, the compound solvent), or medium alone for up to 36 h. Bacterial growth was monitored at multiple time points by measuring optical density at 620 nm (OD_620_).

At 24 h, aliquots from each condition were serially diluted and plated on LB agar to determine colony-forming units (CFU/mL).

### 4.6. Quorum Sensing Biosensor Assays

To investigate the impact of MK-8245 on quorum sensing signal production in *P. aeruginosa*, three biosensor mutant strains, *ΔlasI, ΔrhlI* and *ΔpqsA* (kindly provided by Prof. L. Rampioni, Università degli Studi Roma Tre), were employed [44]. These strains are unable to synthesize their respective autoinducers (3OC12-HSL, C4-HSL, and PQS) but harbor a chromosomally integrated *luxCDABE* reporter operon under the control of QS-responsive promoters. The presence of exogenous autoinducers triggers a regulatory cascade that culminates in luciferase expression and bioluminescence, with signal intensity directly proportional to the concentration of the corresponding molecule.

PAO1 cultures (~1 × 10^6^ CFU/mL) were incubated in LB broth at 37 °C for 24 h in the presence or absence of MK-8245 (40 µM). The compound was pre-dissolved in dimethyl sulfoxide (DMSO, final concentration 0.1%; Merck, Kenilworth, NJ, USA), and control cultures contained an equivalent concentration of DMSO. After incubation, cultures were centrifuged (4000× *g*, 10 min, 4 °C) and cell-free supernatants were obtained by filtration through 0.22 µm pore-size membranes (Millipore, Burlington, MA, USA).

Supernatants were then mixed 1:1 (*v*/*v*) with biosensor cultures (1 × 10^6^ CFU/mL) in white 96-well microplates (Corning, Corning, NY, USA). Bioluminescence was monitored every 5 min for 14.5 h at 37 °C using Varioskan lux multi-mode microplate reader (Thermoscientific, Waltham, MA, USA) with pulsed shaking for 5 s between readings. Luminescence values were normalized to OD_600_ to correct for differences in bacterial growth and the area under the curve (AUC) of each signal was then compared to the control.

To calibrate the biosensors, standard concentrations of C4-HSL, 3-oxo-C12-HSL, and PQS (10 µM, 1 µM, 0.1 µM, 0.01 µM, and non-induced control) were tested. The bioluminescence (LUM) values were normalized by OD_600_, and the AUCs were plotted to create standard curves. Since the relationship between concentration and response was nonlinear, a Hill function was used to fit the data, and the AUCs of the experimental samples were interpolated from these calibration curves.

### 4.7. Effect of MK-8245 on Biofilm Development and Established Biofilms

An overnight culture of *P. aeruginosa* PAO1 grown in LB broth at 37 °C was adjusted to ~10^6^ CFU/mL, and 200 µL of the suspension was added to each well of sterile 96-well polystyrene microtiter plates. Plates were incubated at 37 °C with mild agitation (75 rpm).

Treatments with MK-8245 (40 µM), alone or in combination with Imipenem (MIC_50_), were applied at two different time points (Figure 4A,C):

(i) At inoculation, to evaluate effects on biofilm development;
(ii) After 24 h of incubation, to evaluate effects on pre-formed biofilms.

Following treatment, plates were incubated for an additional 16 h at 37 °C. Biofilm biomass was quantified using the crystal violet staining assay. Briefly, wells were washed twice with sterile PBS to remove planktonic cells, air-dried, and stained with 0.1% (*w*/*v*) crystal violet for 20 min at room temperature. Excess dye was removed by rinsing with distilled water, and the bound dye was solubilized with 30% acetic acid. Absorbance was measured at 570 nm using the Varioskan Lux multi-mode microplate reader (Thermo Fisher Scientific, Waltham, MA, USA).

### 4.8. In Vivo Assays Using Galleria mellonella

To investigate the ability of MK-8245 to control *P. aeruginosa* infection, we used the *Galleria mellonella* model, an invertebrate model with no ethical constraints. These larvae possess innate immune responses and are ideal for rapid in vivo studies [45]. *G. mellonella* larvae were acquired from I.N.E.F. srl (Padova, Italy). Before the assays, larvae were kept in the dark at 15 °C to slow their growth. Only last-instar larvae of similar weight (approximately 300 ± 20 mg) and with no visible signs of melanization or damage were selected for experiments. During the setup of the protocol, one additional control group was used in which the larvae were punctured with the needle in order to dismiss possible deaths related to injection trauma. For the experimental procedures, larvae were randomly assigned to the different experimental groups. Infection was performed by inoculating 10^6^ CFU/larva PAO1. Two hrs later, some larvae were injected with MK-8245 (40 µM) or vehicle (PBS + DMSO 0.1%). Treatments were performed using a 10 µL 26 g Hamilton™ Microliter syringe with an injection volume of 10 µL (Hamilton, Hamilton, ON, Canada). Once injected, larvae were kept at 30 °C during the observation period. Survival was monitored for 48 h. For result interpretation, larvae were considered dead when there was no reaction after placing them face up and touching them, and if the body blackened. The survival outcome was assessed by an investigator blinded to the treatment groups.

### 4.9. Virulence Factor Assays

Dose–response curves were generated to evaluate the concentration-dependent effects of MK-8245 on *P. aeruginosa* virulence factors. PAO1 cultures were treated with increasing concentrations of MK-8245 (5, 10, 20, 40, 80, and 100 µM) in LB medium. Cultures (1 × 10^6^ CFU/mL) were incubated at 37 °C for 24 h under the same conditions used for the individual assays described below. Following incubation, supernatants or cells were processed to quantify pyocyanin, swarming motility, rhamnolipid production, and QS-dependent luminescence according to the protocols reported in the following sections. Results were expressed as a percentage and IC_50_ values were calculated by nonlinear regression (four-parameter logistic model) using GraphPad Prism 10 (GraphPad Software, San Diego, CA, USA).

For subsequent functional assays, metformin (10 mg/mL = 60.4 mM) was included as a positive control, as previously reported in the literature to quorum-sensing inhibitory activity in *P. aeruginosa* [46,47].

#### 4.9.1. Pyocyanin Production

Pyocyanin production was measured according to Ugurlu et al. (2016) [48], with minor modifications. Overnight cultures of *P. aeruginosa* PAO1 or clinical isolates were centrifuged and resuspended in LB broth to ~10^8^ CFU/mL. An inoculum of 10^6^ CFU per well was incubated in LB medium at 37 °C for 24 h, either alone or supplemented with MK-8245. After incubation, cultures were centrifuged, and the supernatants were collected. Chloroform was added at a 3:5 (*v*/*v*) ratio, mixed by inversion, and the chloroform phase was transferred to a clean tube. Acidification with 0.2 M HCl produced a pink-colored solution, which was transferred to a 96-well microtiter plate. Absorbance was measured at 520 nm using a Varioskan™ Lux microplate reader (Thermo Fisher Scientific).

#### 4.9.2. Swarming Motility

Swarming motility was assessed in cultures prepared as described for pyocyanin quantification. Overnight cultures were diluted 1:10, and 10 µL of each dilution was spotted onto swarming agar plates (1% tryptone, 0.5% NaCl, 0.5% agar, and 0.5% filter-sterilized D(+)-glucose; Merck). Plates were incubated upright at 37 °C for 36 h. Swarming migration distances were measured using ImageJ1.x (NIH). Untreated PAO1 cultures served as positive controls.

#### 4.9.3. Rhamnolipid Production

Rhamnolipid production was quantified according to Rasamiravaka et al. (2016) [49], with modifications. PAO1 cultures, treated or untreated with MK-8245, were incubated in LB-MOPS medium at 37 °C for 24 h with agitation (175 rpm). Supernatants (10 mL) were collected by centrifugation (3200× *g*, 5 min, 24 °C) and filtered to remove residual cells. The pH was adjusted to 2–3 with 1 N HCl, followed by extraction with ethyl acetate (1:1, *v*/*v*) for 20 s. The organic phase was evaporated to dryness using a centrifugal vacuum evaporator. Residues were resuspended in 2 mL chloroform and mixed with 200 µL methylene blue solution. After mixing and a 15 min phase separation, the chloroform phase was transferred to a clean tube, acidified with 0.2 N HCl (1:2, *v*/*v*), incubated for 10 min at room temperature, and transferred to a 96-well plate. Absorbance was read at 638 nm.

#### 4.9.4. Lactone Production (AHLs)

To assess the impact of MK-8245 on AHL-mediated signaling, PAO1 cultures (~10^6^ CFU/mL) were incubated in LB broth at 37 °C for 24 h with or without 40 µM MK-8245. Compounds were pre-dissolved in DMSO (0.1%, Merck) when required. In parallel, *Vibrio harveyi* BB120 was grown in Marine Broth at 30 °C for 16 h under agitation and diluted 1:5000 into fresh Autoinducer Bioassay (AB) medium.

For the bioluminescence assay, 90 µL of diluted *V. harveyi* culture was mixed with 10 µL of sterile-filtered PAO1 supernatants (treated or untreated). Mixtures were dispensed into sterile white 96-well plates (Costar, Washington, DC, USA) and incubated overnight at 30 °C. Luminescence was measured with a Varioskan lux multi-mode microplate reader (Thermo Fisher Scientific) and normalized to bacterial growth (OD_620_). Untreated PAO1 supernatants served as positive controls, with luminescence set as 100%.

### 4.10. Data Management and Statistical Analysis

Clinical isolates were obtained as part of an anonymized library of respiratory tract specimens collected at the Clinical Microbiology Laboratory of Padua University Hospital. No patient-identifying information was available, and all samples were handled in compliance with applicable privacy regulations.

All experiments were independently repeated at least three times, with each condition tested in triplicate. Statistical analyses were performed using two-way analysis of variance (ANOVA), followed by Bonferroni’s post hoc test for multiple comparisons in GraphPad Prism (version 8.0). A *p*-value ≤ 0.05 was considered statistically significant.

For survival assays in *Galleria mellonella*, Kaplan–Meier survival curves were generated, and statistical significance was determined using the log-rank (Mantel–Cox) test. Endpoint survival at 48 h was additionally compared using Fisher’s exact test.

## Figures and Tables

**Table 1:** List of compounds used in this study, including CAS numbers, catalog identifiers, and reported purity values. All compounds were purchased from MedChemExpress (MCE).

Product Name	CAS Number	Purity	Catalog Number
Cinaciguat	329773-35-5	99.12%	HY14181A
Tafenoquine (Succinate)	106635-81-8	98.97%	HY-111529A
Idebenone	58186-27-9	99.45%	HY-N0303
Nefazodone (hydrochloride)	82752-99-6	99.03%	HY-B1396
Azaperone	1649-18-9	98.88%	HY-B1470
Rupatadine (Fumarate)	182349-12-8	99.27%	HY-13511A
Lumateperone (Tosylate)	1187020-80-9	99.51%	HY-19733
Urapidil	34661-75-1	99.08%	HY-B0716
Sarpogrelate (hydrochloride)	135159-51-2	99.39%	HY-10564
Istradefylline	155270-99-8	98.94%	HY-10888
Pantethine	16816-67-4	99.22%	HY-B1028
Fluphenazine decanoate	5002-47-1	99.36%	HY-B1904
Etofenamate	30544-47-9	99.10%	HY-17361
Dalcetrapib	211513-37-0	98.99%	HY-14950
Trazodone (hydrochloride)	25332-39-2	99.48%	HY-B0478
Aliskiren	173334-57-1	99.05%	HY-12177
MK-8245	1030612-90-8	99.04%	HY-13070

**Table 2:** Primer sequences and annealing temperatures used for qRT-PCR analysis of quorum-sensing genes in *Pseudomonas aeruginosa.* The housekeeping gene *proC* was used as an internal reference for normalization.

Gene	Primers Used for qRT-PCR	Annealing Temp (°C)
*proC (HK)*	fw 5′-CAGGCCGGGCAGTTGCTGTC-3′	60 °C
	rv 5′-GGTCAGGCGCGAGGCTGTCT-3′	
*rhlI*	fw 5′-AAGGACGTCTTCGCCTACCT-3′	60 °C
	rv 5′-GCAGGCTGGACCAGAATATC-3′	
*rhlR*	fw 5′-CATCCGATGCTGATGTCCAACC-3′	60 °C
	rv 5′-ATGATGGCGATTTCCCCGGAAC-3′	
*rhlA*	fw 5′-TGGCCGAACATTTCAACGT-3′	60 °C
	rv 5′-GATTTCCACCTCGTCGTCCTT-3′	

## Data Availability

All data are reported in the results section of the Manuscript.

## Supplementary Materials

The following supporting information can be downloaded at: https://www.mdpi.com/article/10.3390/antibiotics14111116/s1, Figure S1. Biosensors calibration curve. Figure S2. Dose–response analysis of MK-8245 on virulence-associated phenotypes of *Pseudomonas aeruginosa* PAO1. Table S1. Half-maximal inhibitory concentration (IC_50_) values of MK-8245 for quorum-sensing–regulated virulence traits in *Pseudomonas aeruginosa* PAO1.
