# Supplementary material for: Repurposing MK-8245 as a Quorum Sensing Inhibitor to Suppress Virulence and Potentiate Antibiotic Activity in Pseudomonas aeruginosa
Source: Antibiotics (Basel). 2025 Nov 5;14(11):1116. doi: 10.3390/antibiotics14111116 (PMC12649364; doi:10.3390/antibiotics14111116)
Supplement: Supplementary file 1 [file antibiotics-14-01116-s001.zip › antibiotics-3945254-supplementary.pdf]

Supplementary Informations

Figures

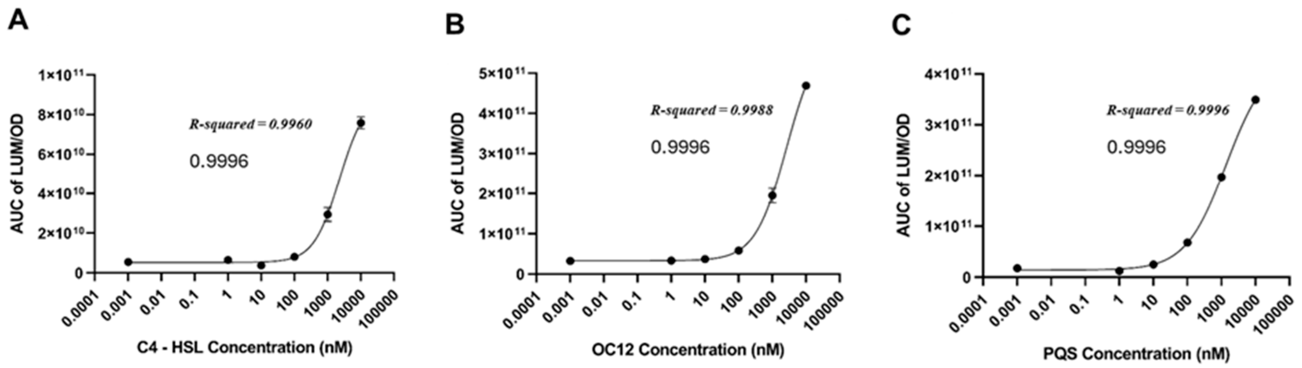

Figure S1. Biosensors calibration curve. A set of standard concentrations (10uM, 1uM, 0.1uM, 0.01uM, Non induced/LB only) of C4-HSL (A), 3-Oxo-C12-HSL (B) and PQS (C) were used to build the standard curves by normalising the bioluminescence (LUM) signal originated from each concentration by the growth of the biosensor (OD at 600 nm). Then, the area under the curve (AUC) was calculated for the resulting LUM/OD. To construct the standard curves, we employed a Hill function since the standard concentrations vs AUC of LUM/OD values are nonlinear, as illustrated in the Figures below. The AUC of LUM/OD values of the samples were then interpolated from those calibration curves.

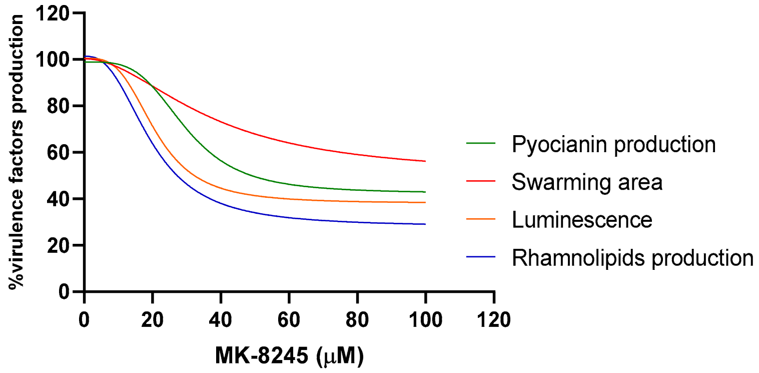

Figure S2. Dose–response analysis of MK-8245 on virulence-associated phenotypes of *Pseudomonas aeruginosa* PAO1. PAO1 cultures were treated with increasing concentrations of MK-8245 (5, 10, 20, 40, 80, 100 μM) in LB medium. The production of pyocyanin, swarming motility, rhamnolipids, and QS-dependent luminescence was quantified after 24 h of incubation. Data are expressed as percentage of the not treated (set as 100%). Curves were fitted using a four-parameter logistic model (GraphPad Prism 10).

Tables

	IC_{50} (µM)
Pyocyanin production	58.4
Swarming area	Not determined
Luminescence	35
Rhamnolipids production	29.2
IC_{50} mean	40.9

Table S1. Half-maximal inhibitory concentration (IC_{50}) values of MK-8245 for quorum-sensing–regulated virulence traits in *Pseudomonas aeruginosa PAO1.* IC_{50} values were determined from nonlinear regression (four-parameter logistic model) of concentration–response curves. MK-8245 inhibited pyocyanin, luminescence, and rhamnolipid production in a concentration-dependent manner, with a mean IC_{50} of 40.9 µM. The IC_{50} for swarming motility could not be reliably determined.
